# Analysis of Serum Metabolomics in Rats with Osteoarthritis by Mass Spectrometry

**DOI:** 10.3390/molecules26237181

**Published:** 2021-11-26

**Authors:** Jingtong Zhao, Meng Liu, Tongfei Shi, Mohan Gao, Yuqian Lv, Yawei Zhao, Jing Li, Ming Zhang, Hansi Zhang, Fengying Guan, Kan He, Li Chen

**Affiliations:** 1 Department of Pharmacology, College of Basic Medical Sciences, Jilin University, Changchun 130061, China; Zhaojt19@mails.jlu.edu.cn (J.Z.); liumeng20@mails.jlu.edu.cn (M.L.); gaomh18@mails.jlu.edu.cn (M.G.); Lvyq19@mails.jlu.edu.cn (Y.L.); zhaoyawei@jlu.edu.cn (Y.Z.); lijing@jlu.edu.cn (J.L.); zhangming99@jlu.edu.cn (M.Z.); zhanghansi@jlu.edu.cn (H.Z.); guanfy@jlu.edu.cn (F.G.); 2 Institutes for Life Sciences, School of Biomedical Sciences and Engineering, South China University of Technology, Guangzhou International Campus, Guangzhou 510006, China; shitf17@mails.jlu.edu.cn; 3 National Engineering Research Center for Tissue Restoration and Reconstruction, South China University of Technology, Guangzhou 510006, China; 4 School of Nursing, Jilin University, Changchun 130020, China

**Keywords:** osteoarthritis, metabolomics, LC/MS, metabolic pathway

## Abstract

Osteoarthritis is a common multifactorial chronic disease that occurs in articular cartilage, subchondral bone, and periarticular tissue. The pathogenesis of OA is still unclear. To investigate the differences in serum metabolites between OA and the control group, liquid chromatography/mass spectrometry (LC/MS)-based metabolomics was used. To reveal the pathogenesis of OA, 12 SD male rats were randomly divided into control and OA groups using collagenase to induce OA for modeling, and serum was collected 7 days after modeling for testing. The OA group was distinguished from the control group by principal component analysis and orthogonal partial least squares-discriminant analysis, and six biomarkers were finally identified. These biomarkers were metabolized through tryptophan metabolism, glutamate metabolism, nitrogen metabolism, spermidine metabolism, and fatty acid metabolism pathways. The study identified metabolites that may be altered in OA, suggesting a role in OA through relevant metabolic pathways. Metabolomics, as an important tool for studying disease mechanisms, provides useful information for studying the metabolic mechanisms of OA.

## 1. Introduction

Osteoarthritis (OA) is an extremely common multifactorial chronic disease that leads to degeneration of joint cartilage, synovial inflammation and osteophyte formation [1]. With the aging of the global population, the pain and disability caused by OA will cause a huge burden on individuals and the social economy [2,3,4]. The most common site for osteoarthritis to occur is the knee joint [5]. However, the mechanisms by which knee OA occurs are still not fully clear [6]. Typically, OA has been considered a disease caused by mechanical damage [7], but an increasing number of studies consider OA to be a low-grade inflammatory disease [8]. The phenotypes associated with OA and low-grade inflammation are emerging as new research hotspots in terms of treatment, diagnosis, and prognosis.

The concept of metabolomics was first introduced in 1999 [9], and nowadays, metabolomics refers to a technique for studying biological metabolic pathways by qualitatively and quantitatively analyzing the composition of all endogenous metabolites in an organism [10]. Metabolomics is an emerging discipline developed after genomics [11], transcriptomics, and proteomics. Metabolomics analyzes small molecules of endogenous metabolites in organisms to elucidate changes in organismal biology by analyzing differential metabolites. Metabolomics analytical techniques mainly include nuclear magnetic resonance (NMR), liquid chromatography/mass spectrometry (LC/MS), and gas chromatography /mass spectrometry (GC/MS). LC/MS has the advantages of high sensitivity, high selectivity, and a wide range of metabolite detection, which makes it suitable for metabolite analysis, especially for non-targeted metabolomics studies. Metabolomic analysis can be performed using osteoarthritic serum or synovial fluid. Synovial fluid is present only locally, whereas serum is present in the whole body; therefore, the serum is the first choice for studying metabolomics [12].

Several untargeted metabolomics studies on OA have been reported [13,14,15,16]. In our study, we investigated the differences between the metabolites of OA group and normal groups by untargeted metabolomics using LC/MS on their serum. This study aimed to find helpful biomarkers of OA and explore the relationship between its metabolic pathways and disease regulation, with the expectation of achieving a deeper understanding of the mechanisms through which OA develops and finding potential therapeutic approaches.

## 2. Results

### 2.1. Histologic Examination and Biochemical Index

**Figure 1:**
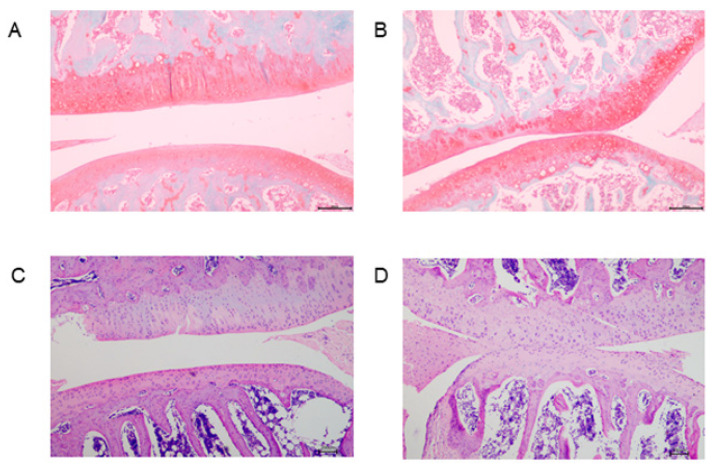
Histologic staining of control group and OA group. (**A**,**B**) Representative safranin O staining for (**A**) control group and (**B**) OA group. (**C**,**D**) Representative HE staining for (**C**) control group and (**D**) OA group. Scale bar, 200 μm.

To verify that the rat OA model was successfully established, we stained rat articular cartilage with Safranin O and histologic examination (HE). As expected, in the OA group, the cartilage content was significantly reduced, the chondrocyte arrangement was disturbed, and the cartilage surface was irregular, which tentatively proved the success of rat modeling (Figure 1).

As shown in Table 1, blood tests and biochemical assays were performed on serum from the OA and control groups. There were no significant changes in bodyweight, white blood cell (WBC), alanine aminotransferase (ALT), and aspartate aminotransferase (AST) between OA and control groups. Serum levels of nitric oxide (NO), prostaglandin estradiol2 (PGE2) and total cholesterol (TC) were obviously increased compared to the control group. However, serum levels of estradiol2 (E2) were obviously decreased.

### 2.2. Multivariate Data Analysis

**Figure 2:**
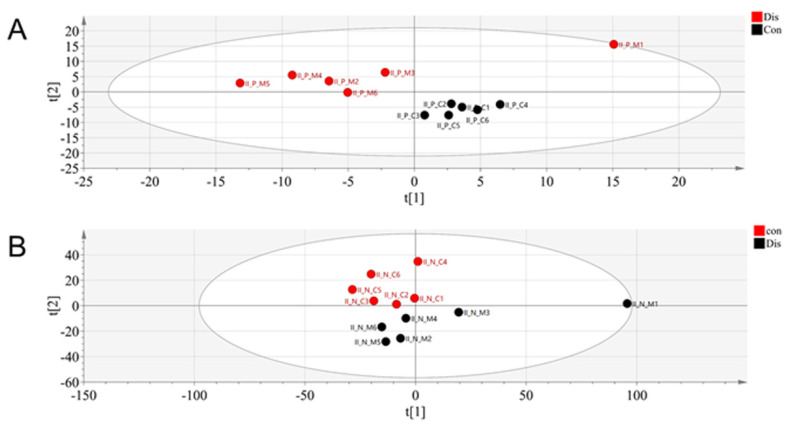
PCA analysis of OA group and control group: (**A**) in positive ion mode and (**B**) in negative ion mode.

**Figure 3:**
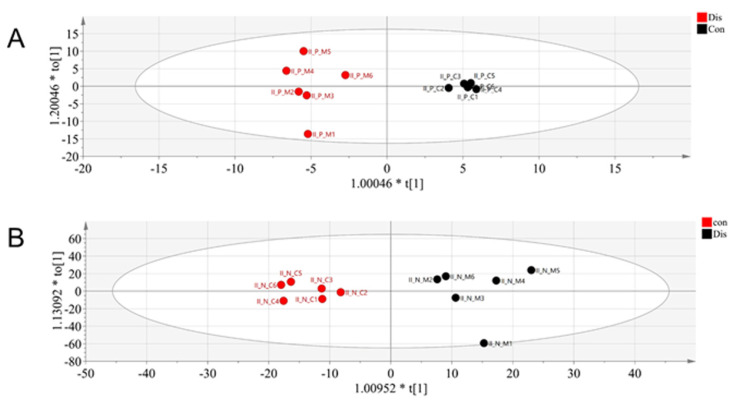
OPLS-DA analysis of OA group and control group: (**A**) in positive ion mode and (**B**) in negative ion mode.

**Figure 4:**
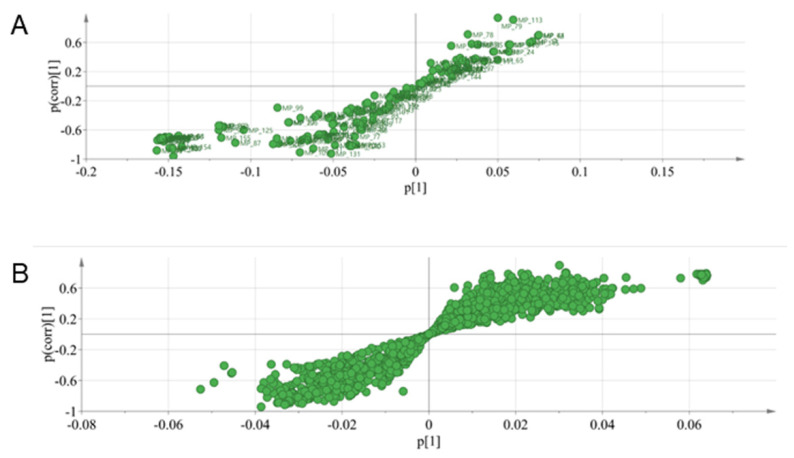
S-plot constructed from serum samples of OA group and control group: (**A**) in positive ion mode and (**B**) in negative ion mode.

We used rapid resolution liquid chromatography/quadrupole-time of light/mass spectrometry (RRLC/Q-TOF/MS) to analyze the serum of the OA and control groups to find out the metabolic differences. The metabolites obtained after mass spectrometry-data-independent acquisition (MS-DIAL) software was imported into Metaboanalyst (http://www.Metaboanalyst.ca/, accessed on 30 March 2021) using a principal components analysis (PCA) model and found that the control and OA groups were distinguishable from each other. As seen in Figure 2, there was a notable variation in the metabolites in the serum of the control rats and the OA rats. The PCA model analysis provided an overall understanding of the metabolite distribution in the serum of each group. To further highlight the distinctions between the groups and facilitate the subsequent hunt for different metabolites, we used orthogonal partial least squares-discriminant analysis (OPLS-DA) to analyze the data. As shown in Figure 3, the control group could be completely separated from the OA group, showing a good model adaptation. In the supervised OPLS-DA model, the control group metabolites in the serum were separated from the OA group metabolites. On this basis, we used S-plots to select the distinguished metabolites between the OA and control groups. S-plot represents the ideal biomarkers with high reliability and a low risk of false positives. Each dot in the S-Plot represents a compound. Samples of two groups distributed in two sides of y axis. The compounds which are located further away from the middle origin and make the greater contribution in the classification will be identified as the potential biomarker (Figure 4).

### 2.3. Identification of Differential Metabolites

Based on the S-plot, there were some ions that indicated distinct variations between the OA and control groups. We used accurate molecular ionic masses to identify six potential biomarkers. In addition, we used databases such as KEGG, HMDB, Massbank, and other databases for analysis of the potential elemental composition, fractional isotope abundance, and unsaturation of the compounds. We have validated the metabolites by using the available standards. The trends of biomarkers, as well as pathways, were listed in Table 2.

## 3. Discussion

Osteoarthritis is a degenerative disease that happens in the articular cartilage, subchondral bone, and periarticular tissue. Factors influencing the occurrence of osteoarthritis are age, obesity, and inflammation. Obesity is one of the most common predisposing factors for OA because of its ability to cause joint overload, leading to cartilage cell death and thus triggering OA. In addition, cytokines secreted by inflammatory mediators can lead to increased cartilage degeneration. However, the pathogenesis of knee OA (KOA) is not clear. Early diagnosis is important because the number of people who develop KOA is increasing due to the aging population as well as to the increase in the number of obese people.

By measuring the bodyweight of rats in the control and OA groups, the two groups were found to be well matched. The successful establishment of the OA model was verified by histologic examinations. According to the biochemical indexes, TC in the OA group was obviously higher than that in the control group, suggesting that the OA group was at risk of obesity. In addition, we found that serum levels of NO and PGE2 were significantly increased in the OA group, while levels of E2, which is associated with the promotion of bone formation, were significantly decreased.

We used a metabolomics approach using LC/MS to collect metabolites and detect metabolic changes between the OA and the control groups. A significant separation between the OA and the control groups was achieved by performing multivariate statistical analysis under the positive and negative ion model, separately. Further analysis by OPLS-DA revealed six metabolites that were tentatively related to metabolic pathways such as nitrogen metabolism, carnitine metabolism, and tryptophan metabolism. We found that these potential biomarkers can demonstrate the occurrence of OA concerning multiple mechanisms, and next, we further investigated the changes in related metabolites.

Tryptophan is a nutritionally essential amino acid that cannot be synthesized in vivo and must be provided through dietary sources. Tryptophan plays a rate-limiting role in protein synthesis, and its main role in the body is as a component of protein synthesis [17]. Tryptophan metabolism is expressed at different levels in rheumatoid arthritis (RA) and OA and can be used to differentiate between RA and OA [18]. L-tryptophan plays an essential role in various chronic inflammatory diseases in humans and is associated with the microenvironment of chronic inflammation in OA joints. In this study, it was shown that L-tryptophan levels were statistically clearly increased in the OA group in comparison to the control group. Therefore, an increase in serum tryptophan concentration may indicate risk of having OA.

The evolution and progression in OA are associated not only with inflammation but also with alterations in amino acid metabolism, such as those of the arginine family of amino acids and related metabolites, such as γ-aminobutyric acid [19]. γ-aminobutyric acid can affect OA progression by inhibiting NF-κB activation [20]. γ-aminobutyric acid was found to be abnormally increased in the serum of OA rats by metabolomics, suggesting that it could serve as a potential metabolic marker for chronic joint pain produced by OA.

In the current study, elevated levels of carbamate were found in OA rat serum. It has been shown that the level of carbamic acid is much higher in patients with osteoarthritis than in normal subjects. Carbamic acid is involved in nitrogen metabolism [21], and inflammatory reactive nitrogen allows for elevated concentrations of basal formic acid and increased oxidative stress [22]. OA as a chronic inflammatory condition is mostly accompanied by acidosis [23], which leads to an increase in acidic metabolites such as butyric acid and stearic acid [24]. Intracellular accumulation of stearic acid can activate inflammatory signaling pathways, release cytokines, and lead to endoplasmic reticulum stress-mediated apoptosis [25]. By metabolomics, a considerable amount of stearic acid levels was found to be increased in the OA group in comparison to the control group.

Arginine has anti-inflammatory and antioxidant properties and is a prerequisite for the syncretization of many molecules [26]. Arginine can contribute to inflammation-related diseases including osteoarthritis [27]. It has been shown that patients with osteoarthritis have reduced arginine concentrations, which may promote the progression of osteoarthritis [28,29]. The competing metabolic pathways of arginase (ARG) and NO synthase (NOS) utilize arginine as a substrate. Arginase produces L-ornithine, which is further metabolized to form proline. Proline is capable of enriching collagenase which leads to fibrosis [30]. L-arginine is used to produce NO through the action of NOS [31]. NO is destructive in mediating inflammatory responses and apoptosis, inhibiting collagen and proteoglycan synthesis and activating matrix metalloproteinases [32,33]. In the present study, the level of L-arginine in OA serum was significantly reduced. Therefore, low concentrations of L-arginine may serve as an indicator of having OA.

L-carnitine is a molecule capable of participating in fatty acid metabolism in mitochondria [34]. Carnitine can cross the mitochondrial membrane to form long-chain acetyl carnitine esters, and carnitine palmitoyltransferase I and carnitine palmitoyltransferase II can transport it [35,36]. L-carnitine is also a β-oxidation cofactor that stabilizes acetyl CoA and coenzyme A in the mitochondrial inner membrane [37,38]. Studies have now demonstrated that L-carnitine influences the metabolism of osteoblasts in vitro and in vivo [39]. In this study showed a significant increase in L-carnitine levels in the OA group versus the control group. Therefore, increased serum L-carnitine concentrations may play an essential role in OA.

Mickiewicz et al. used ^1^H-NMR to detect metabolites in synovial fluid in sheep [40]. They observed that the concentrations of isobutyrate and glucose were higher than in the healthy group. Surowiec et al. found that, when compared to control group, the arginine in the plasma had decreased [11]. Zhang et al. classified OA phenotypes via metabolomic analyses and found that different expressions of acylcarnitines can define a specific phenotype [15]. In our study, we found similar results.

## 4. Materials and Methods

### 4.1. Animal Model Induction and Specimen Collection

The collagenase-induced osteoarthritis (CIOA) model was established according to the protocol in the literature [19]. Healthy six- to eight-week-old male SD rats were purchased through Changchun Yisi Experimental Animal Technology. All of the rats were maintained in the animal facility of the College of Basic Medical Sciences, Jilin University. All rats were acclimatized to the laboratory environment one week before the experiments. All rats were randomly divided into the OA group (*n* = 6) and the control group (*n* = 6). Rats were given anesthesia with 2.5% isoflurane. Briefly, collagenase was dissolved in 0.9% sterile saline, and osteoarthritis was induced by 50 μL (50 unit) injection into the right knee joint. The injections were given on the first and fourth days of the experiment. On day 7, rats were killed for the collection of serum. Serum was separated after collection and stored at −80 °C until processing.

### 4.2. Histological Analysis

Collection from the left knee joint of rats was carried out, and then fixed in 10% buffered formalin. The joints were decalcified and then paraffin-embedded. Then, the joints were stained with safranin O and hematoxylin-eosin (HE).

### 4.3. Blood Test and Biochemical Assay

Bodyweight measurements were performed on all rats. Blood tests and biochemical assays were measured for serum levels of WBC, ALT, AST, NO, PGE2, E2, and TC.

### 4.4. Metabolomics Analysis

We adopted the following protocol for the LC/MS untargeted metabolomics study of all serum samples: Before sample detection, the serum samples were melted at room temperature; 400 μL of methanol was added to 50 μL of the serum samples, shaken vigorously for 30 s, and then left for 10 min at room temperature; the samples were centrifuged at 3000× *g* for 10 min at 4 °C, and the supernatant was taken for mass spectrometry measurement.

LC/MS-based metabolomics analysis has been described previously [37]. Briefly, 5 µL of the samples were injected into an Agilent 1200 series RRLC equipped with the column (AccucoreC18, 2.6 μ, 100 × 2.1 mm; Thermo, Waltham, MA, USA). The chromatography was set at 35 °C during the analysis. The solvent A in the mobile phase was 0.1% formic acid aqueous solution, and B was 0.1% formic acid in acetonitrile. A gradient elution program was performed as follows: 0 min, 90% solvent A; 5 min, 70% solvent A; 10 min, 20% solvent A; 12 min, 15% solvent A; 15 min, 5% solvent A; 18 min, 5% solvent A; 20 min, 90% solvent A; 25 min, 90% solvent A. The flow rate was set at 0.4 mL/min. The total run time was 25 min. 

An Agilent 6520 Q-TOF mass spectrometer (Agilent, Santa Clara, CA, USA) with an electrospray ionzation (ESI) source was used to perform the MS analysis. The mass scan was performed in the range of 80 *m/z* to 1000 *m/z*. Positive and negative modes were used to obtain data by optimizing the parameters: gas temperature, 350 °C; nebulizer, 40 psi; dry gas flow, 12 L/min; intrathecal gas temperature, 350 °C; intrathecal gas flow, 12 L/min.

### 4.5. Univariate and Multivariate Statistical Analysis

Peak identification and peak area normalization were performed on all data using MS-DIAL and Mass Profiler Professional (MPP, Agilent Technologies, Santa Ckara, CA, USA) software. The data were chromatographed for peak identification and matching as well as peak area normalization. The data were stored in files and imported into the SIMCA-P11.0 software package (Umetrics, Umea, Sweden) and multidimensional statistical analysis was performed using unsupervised PCA. In order to strengthen the differences between the OA group and the control group, a supervised pattern recognition method was further used for multidimensional statistical analysis, and OPLS-DA was established for the analysis.

### 4.6. Biomarker and Pathway Analysis

PCA and OPLS-DA analyses were used to compare the metabolite differences between the OA group and the control group. The metabolic pathways that were involved in the discrepant metabolites were analyzed using the KEGG, HMDB, and Massbank databases.

KEGG: http://www.kegg.com (accessed on 12 March 2021)

HMDB: http://www.hmdb.ca/ (accessed on 20 March 2021)

Massbank: http://www.massbank.jp (accessed on 30 March2021)

## 5. Conclusions

We investigated the metabolic differences between OA and controls by using a metabolomics approach with LC/MS. We identified potential biomarkers and showed metabolic disturbances in OA concerning amino acid metabolism, nitrogen metabolism, and carnitine metabolism. Based on the results of our study, we believe that the LC/MS metabolomics approach is an effective instrument for studying the mechanisms for OA and provides helpful information for learning about the diagnosis and treatment of OA.

## Figures and Tables

**Table 1:** Comparison of blood index and biochemical index between control group and OA group.

Characteristic	Controls (*n* = 6)	OA (*n* = 6)
weight (g)	431.7 ± 14.50	421.9 ± 12.19
WBC (/10^9^ × L^−1^)	10.16 ± 1.93	9.88 ± 2.67
ALT (U/L)	47.71 ± 4.26	48.94 ± 6.81
AST (U/L)	116.91 ± 14.05	117.12 ± 11.98
NO (μmol/L)	7.45 ± 1.21	8.69 ± 0.52 *
PGE2 (μmol/L)	7.69 ± 1.60	11.83 ± 2.87 *
E2 (ng/L)	20.07 ± 6.79	10.16 ± 4.42 *
TC (mmol/L)	1.18 ± 0.44	1.96 ± 0.58 *

All data are given as mean ± SD. * *p* < 0.05 OA group compared with the control group.

**Table 2:** Trends in identification results and biomarkers.

Metabolites	Theory (*m/z*) (HMDB)	Observed (*m/z*)	Mass Error Theory vs Observed (ppm)	Observed Retention Time (min)	Commercial Standard (*m/z*)	Mass Error Observed vs Commercial Standard (ppm)	Commercial Standard Retention Time (min)	OA vs Control Group	Pathway
**Positive Ion Mode**									
l-Tryptophan	205.0977	205.0981	2.0	8.4	205.0984	1.5	8.4	Increase	Tryptophan metabolism
γ-Aminobutyric acid	104.0711	104.0709	1.9	7.3	104.0706	2.9	7.3	Increase	Glutamate metabolism
Carbamic acid	62.0242	62.0241	1.6	10.2	62.0242	1.6	10.2	Increase	Nitrogen metabolism
l-Arginine	175.1195	175.1193	1.1	11.3	175.1196	1.7	11.3	Decrease	Arginine metabolism
l-Carnitine	162.113	162.1128	1.2	5.8	162.1131	1.2	5.8	Increase	Fatty acid metabolism
**Negative Ion Mode**									
Stearic acid	283.2637	283.2639	0.7	2.1	283.2638	0.7	2.1	Increase	Fatty acid biosynthesis

## Data Availability

The data generated or analyzed during the study are included in the article.

## Abbreviations

ALT	alanine aminotransferase
ARG	arginase
AST	aspartate aminotransferase
CIOA	collagenase-induced osteoarthritis
E2	estradiol2
GC/MS	gas chromatography /mass spectrometry
HE	histologic examination
KOA	knee osteoarthritis
LC/MS	liquid chromatography/mass spectrometry
MS-DIAL	mass spectrometry-data-independent acquisition
NMR	nuclear magnetic resonance
NO	nitric oxide
NOS	nitric oxide synthase
OA	osteoarthritis
OPLS-DA	orthogonal partial least squares-discriminant analysis
PCA	principal components analysis
PGE2	prostaglandin estradiol2
RA	rheumatoid arthritis
RRLC/Q-TOF/MS	liquid chromatography/quadrupole-time of light/mass spectrometry
TC	total cholesterol
WBC	white blood cell

## References

[1] Li H., Guo H., Lei C., Liu L., Xu L., Feng Y., Ke J., Fang W., Song H., Xu C. (2019). Nanotherapy in Joints: Increasing Endogenous Hyaluronan Production by Delivering Hyaluronan Synthase 2. Adv. Mater..

[2] Jeong J., Bae K., Kim S.G., Kwak D., Moon Y.J., Choi C.H., Kim Y.R., Na C.S., Kim S.J. (2018). Anti-osteoarthritic effects of ChondroT in a rat model of collagenase-induced osteoarthritis. BMC Complement Altern Med..

[3] Lampropoulou-Adamidou K., Lelovas P., Karadimas E.V., Liakou C., Triantafillopoulos I.K., Dontas I., Papaioannou N.A. (2014). Useful animal models for the research of osteoarthritis. Eur J. Orthop. Surg Traumatol..

[4] Hunter D.J., Bierma-Zeinstra S. (2019). Osteoarthritis. Lancet.

[5] Johnson V.L., Hunter D.J. (2014). The epidemiology of osteoarthritis. Best Pract. Res. Clin. Rheumatol..

[6] Conde J., Scotece M., Gomez R., Lopez V., Gomez-Reino J.J., Gualillo O. (2011). Adipokines and osteoarthritis: Novel molecules involved in the pathogenesis and progression of disease. Arthritis..

[7] Sharma L. (2021). Osteoarthritis of the Knee. N. Engl. J. Med..

[8] Al Haj Ahmad R.M., Al-Domi H.A. (2017). Complement 3 serum levels as a pro-inflammatory biomarker for insulin resistance in obesity. Diabetes Metab. Syndr..

[9] Nicholson J.K., Lindon J.C., Holmes E. (1999). ‘Metabonomics’: Understanding the metabolic responses of living systems to pathophysiological stimuli via multivariate statistical analysis of biological NMR spectroscopic data. Xenobiotica.

[10] Fiehn O. (2002). Metabolomics––The link between genotypes and phenotypes. Plant Mol. Biol..

[11] Weng R., Shen S., Tian Y., Burton C., Xu X., Liu Y., Chang C., Bai Y., Liu H. (2015). Metabolomics Approach Reveals Integrated Metabolic Network Associated with Serotonin Deficiency. Sci. Rep..

[12] Liakh I., Sledzinski T., Kaska L., Mozolewska P., Mika A. (2020). Sample Preparation Methods for Lipidomics Approaches Used in Studies of Obesity. Molecules.

[13] Adams S.B., Setton L.A., Nettles D.L. (2013). The role of metabolomics in osteoarthritis research. J. Am. Acad. Orthop. Surg..

[14] McIlwraith C.W., Kawcak C.E., Frisbie D.D., Little C.B., Clegg P.D., Peffers M.J., Karsdal M.A., Ekman S., Laverty S., Slayden R.A. (2018). Biomarkers for equine joint injury and osteoarthritis. J. Orthop. Res..

[15] Zhang W., Likhodii S., Zhang Y., Aref-Eshghi E., Harper P.E., Randell E., Green R., Martin G., Furey A., Sun G. (2014). Classification of osteoarthritis phenotypes by metabolomics analysis. BMJ Open.

[16] Richard D.M., Dawes M.A., Mathias C.W., Acheson A., Hill-Kapturczak N., Dougherty D.M. (2009). L-Tryptophan: Basic Metabolic Functions, Behavioral Research and Therapeutic Indications. Inter. J. Tryptophan Res..

[17] Li Y., Xiao W., Luo W., Zeng C., Deng Z., Ren W., Wu G., Lei G. (2016). Alterations of amino acid metabolism in osteoarthritis: Its implications for nutrition and health. Amino Acid..

[18] Mailankot M., Staniszewska M.M., Butler H., Caprara M.H., Howell S., Wang B. (2009). Indoleamine 2,3-dioxygenase overexpression causes kynurenine-modification of proteins, fiber cell apoptosis and cataract formation in the mouse lens. Lab. Invest..

[19] Huang S., Mao J., Wei B., Pei G. (2015). The anti-spasticity drug baclofen alleviates collagen-induced arthritis and regulates dendritic cells. J. Cell. Physiol..

[20] Blanco F.J., Valdes A.M., Rego-Pérez I. (2018). Mitochondrial DNA variation and the pathogenesis of osteoarthritis phenotypes. Nat. Rev. Rheumatol..

[21] Senol O., Gundogdu G., Gundogdu K., Miloglu F.D. (2019). Investigation of the relationships between knee osteoarthritis and obesity via untargeted metabolomics analysis. Clin. Rheumatol..

[22] Levick J.R. (1990). Hypoxia and acidosis in chronic inflammatory arthritis; relation to vascular supply and dynamic effusion pressure. J. Rheumatol..

[23] Collins J.A., Moots R.J., Winstanley R., Clegg P.D., Milner P.I. (2013). Oxygen and pH-sensitivity of human osteoarthritic chondrocytes in 3-D alginate bead culture system. Osteoarthr. Cartil..

[24] Morris S.M. (2006). Arginine: Beyond protein. Am. J Clin. Nutr..

[25] Anderson E.K., Hill A.A., Hasty A.H. (2012). Stearic acid accumulation in macrophages induces toll-like receptor 4/2-independent inflammation leading to endoplasmic reticulum stress-mediated apoptosis. Arterioscler. Thromb. Vasc. Biol..

[26] Li J.T., Zeng N., Yan Z.P., Liao T., Ni G.X. (2021). A review of applications of metabolomics in osteoarthritis. Clin. Rheumatol..

[27] Ohnishi A., Osaki T., Matahira Y., Tsuka T., Imagawa T., Okamoto Y., Minami S. (2013). Correlation of plasma amino acid concentrations and chondroprotective effects of glucosamine and fish collagen peptide on the development of osteoarthritis. J. Vet. Med. Sci..

[28] Tootsi K., Vilba K., Märtson A., Kals J., Paapstel K., Zilmer M. (2020). Metabolomic Signature of Amino Acids, Biogenic Amines and Lipids in Blood Serum of Patients with Severe Osteoarthritis. Metabolites.

[29] Rockel J.S., Kapoor M. (2018). The Metabolome and Osteoarthritis: Possible Contributions to Symptoms and Pathology. Metabolites.

[30] Abramson S.B., Amin A.R., Clancy R.M., Attur M. (2001). The role of nitric oxide in tissue destruction. Best Pract. Res. Clin. Rheumatol..

[31] Carlson A.K., Rawle R.A., Adams E., Greenwood M.C., Bothner B., June R.K. (2018). Application of global metabolomic profiling of synovial fluid for osteoarthritis biomarkers. Biochem. Biophys. Res. Commun..

[32] Abramson S.B. (2008). Nitric oxide in inflammation and pain associated with osteoarthritis. Arthritis Res. Ther..

[33] Pekala J., Patkowska-Sokoła B., Bodkowski R., Jamroz D., Nowakowski P., Lochyński S., Librowski T. (2011). L-carnitine-metabolic functions and meaning in humans life. Curr. Drug Metab..

[34] Belsky J.B., Wira C.R., Jacob V., Sather J.E., Lee P.J. (2018). A review of micronutrients in sepsis: The role of thiamine, l-carnitine, vitamin C, selenium and vitamin D. Nutr. Res. Rev..

[35] Savic D., Hodson L., Neubauer S., Pavlides M. (2020). The Importance of the Fatty Acid Transporter L-Carnitine in Non-Alcoholic Fatty Liver Disease (NAFLD). Nutrients.

[36] Chapela S.P., Kriguer N., Fernández E.H., Stella C.A. (2009). Involvement of L-carnitine in cellular metabolism: Beyond Acyl-CoA transport. Mini Rev. Med. Chem..

[37] Karlic H., Lohninger A. (2004). Supplementation of L-carnitine in athletes: Does it make sense?. Nutrition.

[38] Stoppoloni D., Politi L., Dalla Vedova P., Messano M., Koverech A., Scandurra R., Scotto d’Abusco A. (2013). L-carnitine enhances extracellular matrix synthesis in human primary chondrocytes. Rheumatol. Inter..

[39] Jia C., Xu H., Xu Y., Xu Y., Shi Q. (2019). Serum metabolomics analysis of patients with polycystic ovary syndrome by mass spectrometry. Mol. Reprod. Dev..

[40] Surowiec I., Arlestig S., Rantapaa-Dahlqvist J. (2016). Metabolite and Lipid Profiling of Biobank Plasma Samples Collected Prior to Onset of Rheumatoid Arthritis. PLoS ONE.

